# Transport Analysis of Anti-Wetting Composite Fibrous Membranes for Membrane Distillation

**DOI:** 10.3390/membranes11010014

**Published:** 2020-12-24

**Authors:** Jingcheng Cai, Zeman Liu, Fei Guo

**Affiliations:** School of Energy and Power Engineering, Key Laboratory of Ocean Energy Utilization and Energy Conservation of Ministry of Education, Dalian University of Technology, No.2 Linggong Road, Dalian 116024, China; wuyouwubu252@foxmail.com (J.C.); liuzm@mail.dlut.edu.cn (Z.L.)

**Keywords:** composite fibrous membrane, membrane distillation, transport analysis, liquid entry pressure, porosity

## Abstract

Composite electrospun fibrous membranes are widely studied for the application of membrane distillation. It is an effective approach to enhance the membrane distillation performance in terms of anti-wetting surface and permeate flux by fabricating composite fibrous membranes (CFMs) with a thin skin layer on a thick supporting layer. In this work, various membranes prepared with different pore sizes and porosities by polyacrylonitrile and polyvinylpyrrolidone were prepared. The membrane characteristics and membrane distillation performance were tested. The mass transfer across the membranes was analyzed experimentally and theoretically in detail. It is shown that the skin layer significantly increases liquid entry pressure of the CFM by 5 times. All the membranes have a similar permeate flux. The permeate flux of membranes is stable at 19.2 ± 1.2 kg/m^2^/h, and the salt rejection ratios remain above 99.98% at 78 ± 1 °C for 11 h. The pore size and porosity of membranes have an insignificant effect on the temperature distribution of membrane. The porosity and pore size of the skin layer have an insignificant effect on the mass transfer process of the CFM. The mass transfer process of the CFM is governed by the supporting layer.

## 1. Introduction

Membrane distillation (MD) is a phase-change membrane-separation process driven by the transmembrane-saturated vapor-pressure difference induced by temperature difference [1,2]. As a gas–liquid barrier, the hydrophobic membrane only allows vapor to go through. Water evaporates on the interface between the feed and membrane and condenses on the cold side of the membrane [3,4]. To prevent membrane wetting and ensure the diffusion space of vapor molecules, commonly used membranes have micron or submicron pore size (0.1–1 μm) [5,6]. The hydrophobic membrane plays two main roles in MD: preventing the contact between the feed and permeate through the membrane pore; and providing efficient transfer space for vapor molecules [7]. Membrane is the core component of MD. The membrane parameters, such as pore size, porosity, and hydrophobicity, are constantly optimized with the development of MD [8,9,10]. The commonly used intrinsic hydrophobic membrane materials include polyvinylidene fluoride (PVDF) [11], polytetrafluoroethylene (PTFE) [12,13], polypropylene (PP) [14], and polyvinylidene fluoride-co-hexafluoropropylene (PVDF-HFP) [15,16]. In the follow-up study, hydrophilic materials such as polyacrylonitrile (PAN) [17] and nylon [18] with hydrophobic surface modification can also be used in MD.

One of the goals of MD is to improve the permeate flux. Membrane with large porosity, small tortuosity, large pore size, and thin thickness can reduce the mass transfer resistance and improve the permeate flux [19,20]. Compared with the membrane prepared by phase inversion or stretching method, the porosity of electrospun membrane can reach more than 90%, and the tortuosity is close to 1 [21,22,23]. Therefore, electrospun membrane can significantly improve the mass transfer coefficient and enhance the MD performance according to the basic mass transfer mechanism [5,24]. Increasing the pore size of the membrane is an effective way to further improve the permeate flux. The increase in membrane pore size reduces liquid entry pressure (LEP) [18,25]. Membranes with low LEP are easy to be wetted leading to a decrease in salt rejection ratio in MD [26]. Therefore, it is difficult to increase the permeate flux and prevent the membrane from being wetted simultaneously in single-layer membranes. Fabrication of composite fibrous membranes (CFMs) with multi-layers is an approach to enhance the MD performance.

CFMs prepared by electrospinning technology have been used in MD to meet different demands [27,28]. Tijing et al. first prepared electrospinning CFMs with PVDF-HFP and PAN to improve permeate flux [29] Hydrophilic and hydrophobic membranes are also used in MD to treat wastewater containing volatile organic compounds and surfactants [30,31]. CFMs with a modified structure (TiO_2_ [32,33], SiO_2_ [34,35,36], carbon nanotubes [37,38], and silver nanoparticle [39,40]) can achieve anti-fouling or anti-bacterial performance. The membranes with different pore sizes of PVDF-HFP skin layer and polyethylene terephthalate (PET) supporting layer can increase the membrane strength and enhance the MD performance [41]. Except for hydrophilic and hydrophobic membranes [42,43], most of the studies on the CFMs are always found in direct contact MD (DCMD) [28,44,45]. Previous studies on CFMs have focused on their characterization and application, the transport behavior in the membranes needs to be further studied.

CFMs were fabricated by electrospinning a thin skin layer with a relatively small fiber diameter on a supporting layer with relatively large fiber diameter in this study. The skin layer was composed of smooth, rough, or beaded fibers. The membrane properties of morphology, porosity, pore size, and LEP were characterized. The air-gap membrane distillation (AGMD) performance in terms of permeate flux and salt rejection ratio as well as the stability of the membrane were tested. The effects of the membrane pore size and porosity on the mass transfer and temperature distribution were theoretical estimated. The influence of skin layer and supporting layer on the MD performance and the transport mechanism of membranes were analyzed. The porosity and pore size of an optimized CFM were proposed after theoretical estimation.

## 2. Materials and Methods

### 2.1. Electrospinning

**Figure 1:**
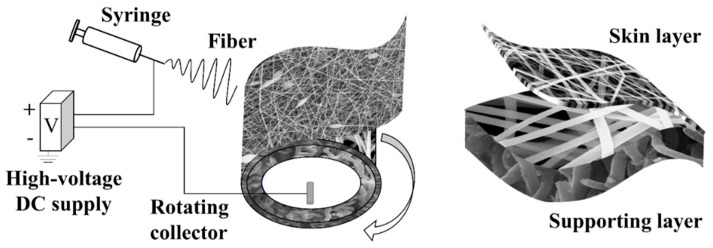
Schematic illustration of an electrospinning setup for manufacturing a CFM.

The polymer solution of PAN (DOW Inc., Midland, MI, USA, *M_w_* = 85,000) and PAN/Polyvinylpyrrolidone (PVP) (Shanghai Aladdin Bio-Chem Technology Co., LTD., Shanghai, China, AR, *M_w_* = 130,000) was prepared with *N*,*N*-dimethylformamide (Shanghai Aladdin Bio-Chem Technology Co., LTD., Shanghai, China, AR, 99.5%) and acetone (Tianjin Kemiou Chemical Reagent Co., Ltd., Tianjin, China, AR, >99.5%) (95/5, wt./wt.) as solvent. To obtain the fiber with different diameters and morphologies, several exploratory tests were carried out. The concentrations of PAN solution were selected as 19 wt.%, 11.5 wt.%, and 7 wt.%. The concentration of PAN/PVP solution was selected as 5.5 wt.%/6 wt.%. The prepared solution was placed on a magnetic stirrer (240 rpm), heated and stirred overnight in a 60 °C water bath, and defoamed at room temperature of 25 ± 1 °C. The relative humidity was 20% ± 5%. The flow rate of the solution was 0.02 mL/min. A rotating collector with a diameter of 10 cm was used as the collector. The rotating speed was 40 rpm. The receiving distance between the jet and the front surface of the rotating collector was 15 cm. The electrospinning parameters of the membrane are shown in Table 1. The electrospinning process of the CFM is shown in Figure 1. First, a supporting layer with a thickness of ~50 μm was electrospun from PAN solution (19 wt.%). Then the skin layer was electrospun on the supporting layer surface. The polymer solutions for the skin layer are shown in Table 2. The solution was quickly switched during the electrospinning process within 5 min. All the membranes were dried at 60 °C for more than 12 h to remove the residual solvent, and then the further tests of microscopic morphology, porosity, water contact angle, and LEP in Section 2.2 as well as MD in Section 2.3 were conducted. Membranes containing PVP were washed in deionized water at 90 °C for ~24 h. The washing process was repeated 3 times.

### 2.2. Membrane Characterization

Scanning electron microscopy (SEM) (Quanta 450, FEI) was used to characterize the membrane morphology. A conductive gold layer was sputtered on the membrane surface by a coating unit (Q150T, Quorum Emitech & Polaron, Laughton, East Sussex, UK) for 90 s before the measurement. Image-Pro Plus 6.0 was used to measure the fiber diameter.

The porosity (*ε*) was measured by the gravimetric method. A rectangular membrane sample with uniform thickness was weighted by an electronic balance and was recorded as *m_m_*. The samples were immersed in isopropanol for 10 min. The membrane was taken out from isopropanol and sandwiched in polyethylene meshes. The excess isopropanol on the membrane surface was wiped off. The wet membrane was weighed as *m_wm_*. The membrane porosity is calculated using the following equation [46]:

(1) $$\varepsilon =\frac{\frac{m_{wm}-m_{m}}{\rho_{\mathrm{IPA}}}}{\frac{m_{wm}-m_{m}}{\rho_{\mathrm{IPA}}}+\frac{m_{m}}{\rho_{p}}}$$

where *ρ*_IPA_ and *ρ_p_* are the densities of the isopropanol and the precursor polymer, respectively. The skin layer porosity can be calculated according to the thickness of the CFM and supporting layer:

(2) $$\varepsilon_{1}=\left(1+\frac{\delta_{2}}{\delta_{1}}\right)\varepsilon_{t}-\frac{\delta_{2}}{\delta_{1}}\varepsilon_{2}$$

where *ε* and *δ* are the porosity and thickness, respectively. The subscripts 1, 2, and *t* represent the skin layer, supporting layer, and CFM, respectively.

All membranes used for contact angle, LEP, and MD tests were transformed into hydrophobic membranes by initiated chemical vapor deposition (iCVD) with 1H,1H,2H,2H–Perfluorodecyl acrylate (PFDA) (Sigma-Aldrich Co. LLC, St. Louis, MO, USA) [17]. The contact angle was measured by a goniometer. Flatten the membrane sample on the sample stage. 2.5 μL of deionized water was randomly dropped on the sample to measure the contact angle. The contact angle tests were repeated 10 times.

**Figure 2:**
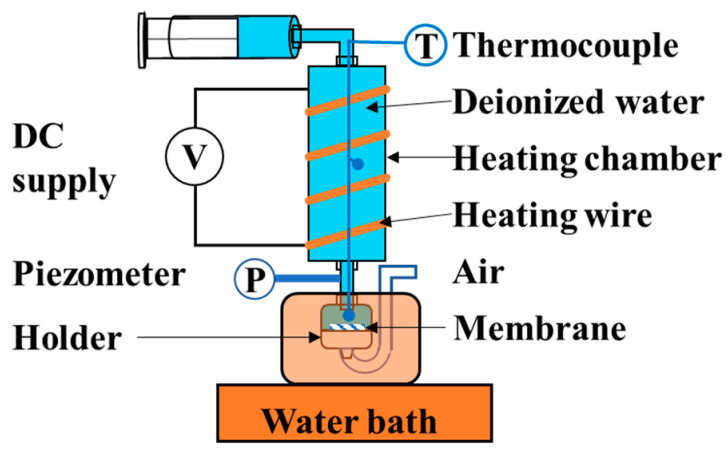
Unit for measuring LEP. Adjust the voltage through the DC supply to control the temperature of the heating wire for controlling the temperature of the heating chamber. One side of the membrane is connected to deionized water and the other side is connected to air.

The LEP tests were carried out with an upgraded unit (see Figure 2) from references [18,47]. The membrane was placed in a stainless steel holder with an effective diameter of 9 mm. A stainless steel mesh was used as membrane support in the holder to ensure that the membrane remained flat under pressure. The heating chamber was filled with deionized water. During the test, the heating chamber was set at the test temperature of 25 °C, 40 °C, 50 °C, 70 °C, and 80 °C, respectively. The temperatures in the heating chamber and holder were measured by thermocouples. The temperature of the water bath was 5 °C higher than that of the heating chamber. The deionized water was pressed into the heating chamber at a constant flow rate (0.3 mL/min) by an injection pump. A piezometer was used to measure the pressure on the membrane. The LEP tests were repeated 3 times.

The pore diameter was estimated by Young-Laplace equation [48]:

(3) d=−4γcosθ/ΔP

where *d* is the pore diameter, Δ*P* is the pressure difference between the liquid and vapor phases (i.e., LEP), *γ* is the liquid surface tension, and *θ* is the intrinsic contact angle of the material.

### 2.3. Membrane Distillation

The MD tests were conducted based on an air-gap membrane distillation (AGMD) system described in detail in the previous studies [17,47]. CuSO_4_ solution (3.5 wt.%, blue color) was used as the feed. The substitution of CuSO_4_ solution for NaCl solution had an insignificant effect on the MD performance [49]. The coolant temperature was kept at 20 ± 1 °C in all the tests. The flow rates of the feed and coolant were 0.8 L/min and 2 L/min, respectively. The circulation pressure of the feed in the MD system is ~15 kPa. The stability of the membranes was tested at 78 °C for 11 h. The permeate concentration was detected by a total dissolved solids (TDS) sensor. The salt rejection ratio was estimated by the TDS of the feed and permeate.

**Figure 3:**
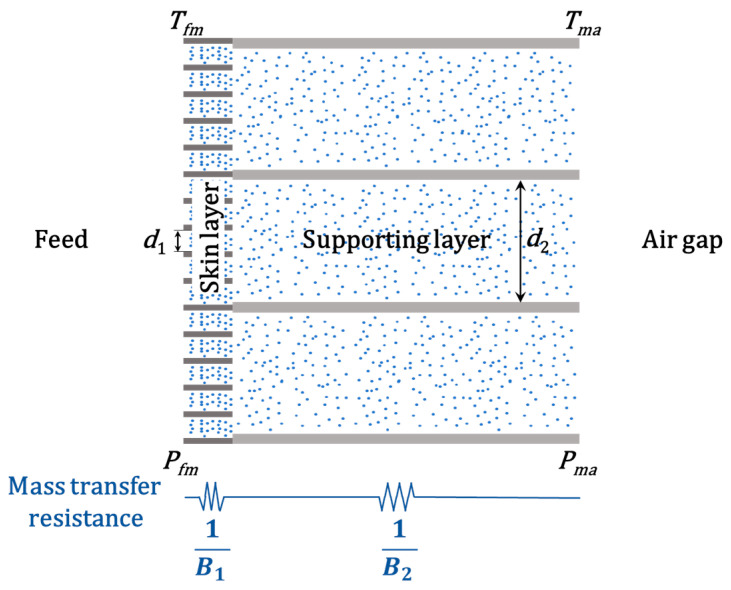
Schematic diagram of the transport behavior of water molecules in the CFM. The blue dots represent water molecules.

To analysis the mass transfer in the MD, permeate flux is estimated by Darcy’s law [50]:

(4) $$N=B_{m}\left(P_{fm}-P_{ma}\right)$$

where *N* is the permeate flux, *P_fm_* is the saturated vapor pressure on the interface between the feed and the membrane, *P_ma_* is the saturated vapor pressure on the interface between the membrane and the air gap (see Figure 3). *P_fm_* and *P_ma_* can be calculated based on the temperature of the corresponding interface (*T_fm_* and *T_ma_*), respectively. *B_m_* is the mass transfer coefficient for the membrane, which is given by [51,52]:

(5) $$\frac{1}{B_{m}}=\frac{1}{B_{1}}+\frac{1}{B_{2}}$$

where *B*_1_ and *B*_2_ are the mass transfer coefficients for the skin layer and supporting layer (see Figure 3). *B*_1_ and *B*_2_ are determined by transition diffusion in this work (Knudsen number: 0.04–0.5) and can be evaluated by the following simplified equation [5,53,54]:

(6) $$\frac{1}{B_{1}}=\frac{\delta_{1}}{\varepsilon_{1}^{2}}\left(\frac{1}{C_{K}d_{1}}+\frac{1}{C_{M}}\right),\;\frac{1}{B_{2}}=\frac{\delta_{2}}{\varepsilon_{2}^{2}}\left(\frac{1}{C_{K}d_{2}}+\frac{1}{C_{M}}\right)$$

where *B* is the mass transfer coefficient, *ε* is the porosity, *d* is the pore diameter, and *δ* is the thickness. The subscripts 1 and 2 represent the skin layer and supporting layer, respectively. *C_K_* and *C_M_* are coefficients related to temperature in Knudsen diffusion and molecular diffusion, respectively [3,5]:

(7) $$C_{K}=2.48\times 10^{-2}{\left(\frac{1}{T}\right)}^{\frac{1}{2}},\;C_{M}=4.09\times 10^{-8}\frac{T^{1.072}}{P_{a}}$$

where *P_a_* is the partial pressure of air in the pore, *T* is the temperature in the pore.

## 3. Results and Discussion

### 3.1. Morphology of the Membrane

**Figure 4:**
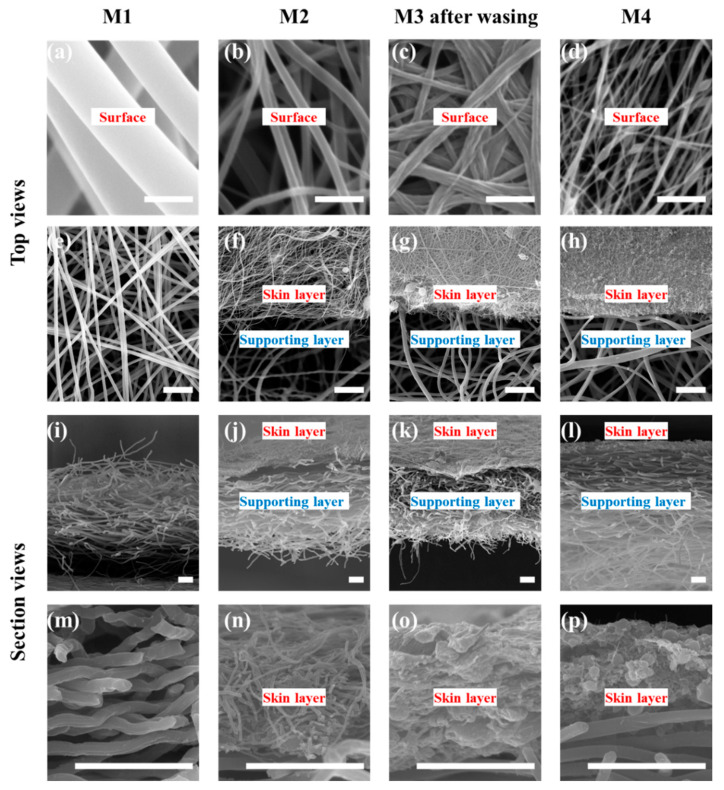
Morphologies of membranes. (**a**–**d**) show the microstructure of the membrane surface. (**e**–**h**) show the top view of the cross-section in the membranes. (**i**–**l**) show the section view of the cross-section in the membranes. (**m**–**p**) are the cross-section of M1 and the skin layer of CFMs. The scale bars of (**a**–**d**) are 1 μm. The scale bars of (**e**–**p**) are 10 μm.

The surface and cross-section of membranes is shown in the SEM images (see Figure 4). The code, thickness, and composition of the membranes are shown in Table 2. The fiber diameters of the supporting layer of the M2, M3, and M4 as well as the membrane M1 are 1.1 ± 0.1 μm. The thickness of M1 electrospun from PAN solution (19 wt.%) is 60 ± 2 μm. The skin layer of M2 is electrospun from PAN solution (11.5 wt.%). The fiber diameter of the skin layer of M2 is 0.2 ± 0.05 μm, and the thickness is ~7 μm. M1 and the skin layer of M2 are composed of fibers with a cylindrical structure and a smooth surface. The skin layer of M3 composed of fibers with a rough surface is electrospun from PAN/PVP solution (5.5 wt.%/6 wt.%). The fiber diameter of the skin layer of M3 after washing is 0.25 ± 0.05 μm, and the thickness is ~9 μm. The skin layer of M4 composed of beaded fibers is electrospun from PAN solution (7 wt.%) with a thickness of ~7 μm. The thickness of the supporting layers is 50 ± 4 μm.

There was no adhesion between fibers in the skin layer of M1 and M2. The fibers in the skin layer of M3 and M4 adhere to each other. The reasons for the adhesion are different. The residual PVP can still be found in the skin layer of M3 by Flourier-transform infrared spectroscopy (FTIR) analysis (see Supporting Information (SI) Figure S1). Thus, in the washing process, PVP adheres to the fiber surface, resulting in the adhesion between the fibers. The adhesion in the skin layer of M4 is due to the slow volatilization of the solvent in the bead due to the smaller specific surface. The bead dissolves the fiber and adheres to it [55]. The fiber diameter (0.19 ± 0.05 μm, see SI Figure S2) of the skin layer of M3 before washing is almost equal to that of M2. After washing, the fiber diameter of the skin layer of M3 increases slightly (see Table 1), and the fiber surface becomes rough.

**Figure 5:**
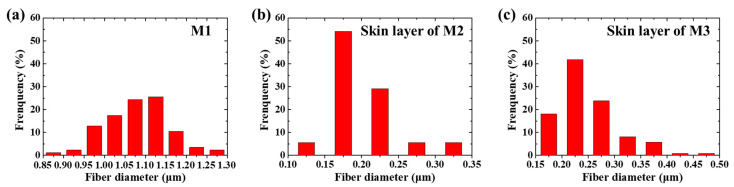
The statistical diagram of the fiber diameter distributions in (**a**) M1, (**b**) skin layer of M2, and (**c**) skin layer of M3.

The fiber diameter distributions of M1, skin layer of M2, and skin layer of M3 are in the ranges of 0.85–1.3 μm, 0.1–0.35 μm, and 0.15–0.5 μm (see Figure 5). The average diameter is shown in Table 1. All the distributions are close to normal. The bead diameter of the beaded fiber is 0.6 ± 0.4 μm. The diameter of the fiber connecting the beads is 0.05 ± 0.02 μm. The experimental data show that the smaller the average fiber diameter, the narrower the fiber diameter distribution. This is because the larger fiber diameter is caused by the higher concentration of the electrospinning solution. The viscoelastic force and surface tension of the electrospinning solution with high concentration are difficult to overcome by the electrostatic force leading to an unstable electrospinning process [21].

The membrane porosity is shown in Table 2. The porosities of the skin layer of M1 and M2 with unadhered fibers are much higher than that of M3 and M4 with adhered fibers. This is because the three-dimensional pore structure of the membrane changes into a two-dimensional structure after the fibers adhere to each other. The free overlap between fibers is converted into fixed adhesion, so space for fibers to move is reduced. For electrospun fibrous membrane with a unadhered three-dimensional open structure, the smaller the fiber diameter, the higher the porosity [18,56].

### 3.2. Wettability of the Membranes

**Figure 6:**
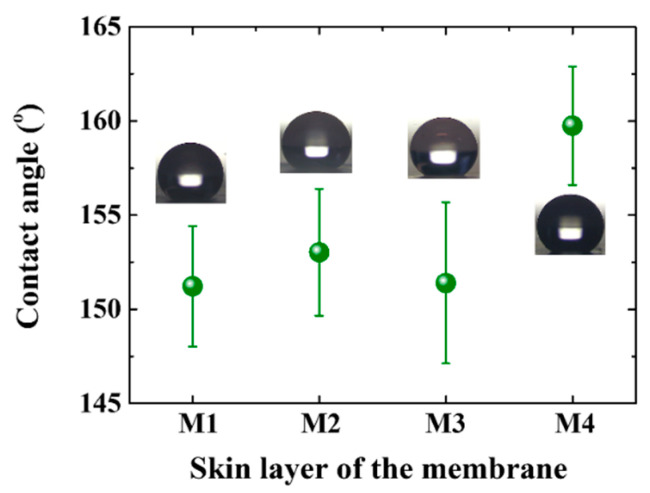
Contact angle of the skin layer of the membrane. The test is carried out at 25 °C. The volume of deionized water is 2.5 μL.

According to Cassie-Baxter theory, the contact angle of the membrane is larger with higher porosity, smaller surface energy, and rougher surface [57]. The porosity of the electrospun fibrous membrane is high, and the surface energy of coating material (PFDA) is 9.3 mN/m [58]. Thus, the contact angles of all the hydrophobic modified membranes are above 150° (see Figure 6). The contact angle of M2 is larger than that of M1 due to the higher skin layer porosity. The low skin layer porosity of M3 decreases its contact angle. The washing process may lead to a rougher surface of the skin layer of M3. Thus, the contact angle of M3 is close to that of M1. The contact angle of M4 is ~160°, which is consistent with the previous study [59]. The rougher surface of the beaded fibrous membrane leads to a smaller contact surface between the droplet and surface [60,61].

**Figure 7:**
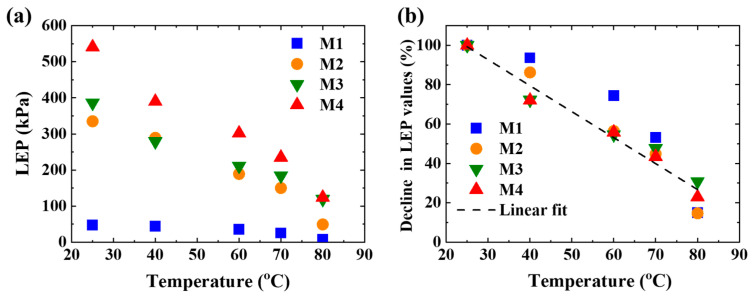
(**a**) LEP of all the membranes at different temperatures. The flow rate is 0.3 mL/min. The test temperature is 25–80 °C. (**b**) The decline rates of LEP at different temperatures.

The LEP of all the membranes at different temperatures is shown in Figure 7a and SI Figure S3. The LEP values at 25 °C of M1, M2, M3, and M4 are 47 kPa, 335 kPa, 386 kPa, and 541 kPa, respectively. According to the Young–Laplace equation, LEP is mainly determined by the membrane pore size, surface energy of test liquid, and surface energy of membrane. The surface energy of the membrane is the same after hydrophobic modification (see contact angle analysis). Deionized water was used for all the tests. Therefore, LEP is only governed by the membrane pore size. For electrospun fibrous membranes, the larger the fiber diameter, the larger the membrane pore size [62]. The fiber diameters of the skin layer of M2, M3, and M4 are less than 1/5 of that of M1 leading to at least 5 times higher LEP at the same temperature. Thus, the LEP can be significantly improved by electrospinning a membrane with a small fiber diameter on the supporting layer.

LEP is also affected by the fiber morphology. The skin layer of M4 is composed of beaded fiber with the smallest diameter of the fiber linking the beads. The bead can be considered to be the expansion of the fiber leading to a further decrease in the membrane pore size. Thus, the LEP value of M4 is highest under the same temperature among M1, M2, M3, and M4. The adsorbed PVP on the fiber surface in the skin layer of M3 decreases the membrane pore size. The LEP value of M3 is higher compared to that of M2.

The ratio of LEP at different temperatures to that at 25 °C is plotted in Figure 7b. The ratio of LEP almost decreases linearly with a slope of ~0.013 (1/°C). LEP decreases by 70%–80% with the increase of temperature from 25 °C to 80 °C. The surface tension of water only decreases by 14% under the same temperature difference [63]. The decrease in LEP requires further investigations.

### 3.3. Membrane Distillation

**Figure 8:**
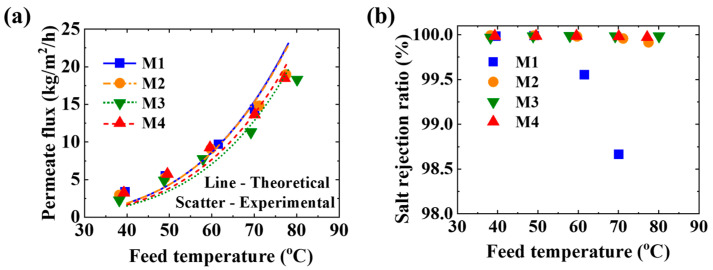
MD performance in terms of (**a**) permeate flux (**b**) salt rejection ratio under different temperatures.

As shown in Figure 8a, the permeate flux increases from 2 kg/m^2^/h to 20 kg/m^2^/h with the feed temperature from 40 °C to 80 °C. When the feed temperature is higher than 50 °C, the salt rejection ratio for M1 decreases. The permeate of MD with M1 is blue under the feed temperature of 80 °C. This indicates the membrane was wetted. As shown in Figure 8b, the salt rejection ratios for M2, M3, and M4 remain higher than 99.98%. The permeate is colorless and transparent with TDS less than 10 ppm. This is because the LEP values of all the membranes (40 kPa at the feed temperature ≤ 40 °C) are higher than the circulation pressure of the feed (see Section 2.3). When the feed temperature is 80 °C, the LEP of M2, M3, and M4 is still higher than 40 kPa, while that of M1 is only 10 kPa. Therefore, by electrospinning a thin layer with small diameter fiber on its surface, the membrane with a relatively large pore size can be applied to MD.

**Figure 9:**
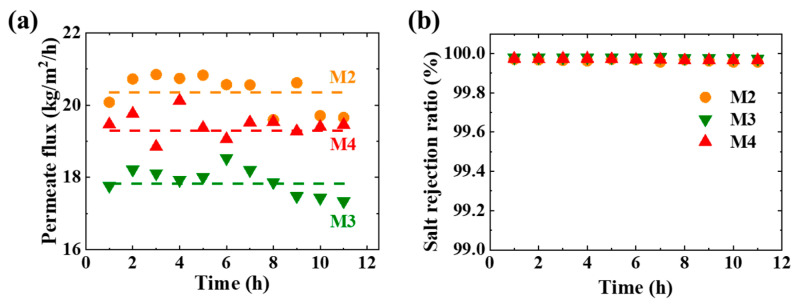
(**a**) Permeate flux and (**b**) salt rejection ratio for the CFMs in MD under extreme conditions. The feed temperature is 78 ± 1 °C. The test is running for 11 h.

The stability of M2, M3, and M4 is tested for 11 h under the feed temperature of 78 ± 1 °C. As shown in Figure 9a, the permeate fluxes of M2, M3, and M4 are stable at 17.9 ± 0.4, 19.4 ± 0.3, and 20.4 ± 0.5 kg/m^2^/h, respectively. The salt rejection ratios for M2, M3, and M4 in MD are always above 99.98%. The permeate flux is almost independent of the operation time. This indicates that the properties of the M2, M3, M4, and transport behavior in MD remain unchanged. The CFMs have good chemical stability, mechanical stability, and thermal stability.

The CFM combines the advantages of the supporting layer and the skin layer. The supporting layer has high electrospinning efficiency and low mass transfer resistance. The skin layer has a large LEP at the cost of long electrospinning time and complicated manufacturing process. When the electrospinning time is 2 h, the PAN solution (19 wt.%) can be electrospun into a membrane with an area of 20 cm × 31.4 cm and a thickness of ~50 μm. The thickness of the membrane electrospun with a PAN solution (11.5 wt.%) or PAN/PVP solution (5.5 wt.%/6 wt.%) is only 30 μm under the same conditions. The thickness of the membrane electrospun with PAN (7 wt.%) solution is only 3 μm under the same electrospinning time. As the surface tension of PAN solution (7 wt.%) is too high, the solution drops between the needle and the collector without electrospun into fibers.

### 3.4. Permeation Analysis

**Figure 10:**
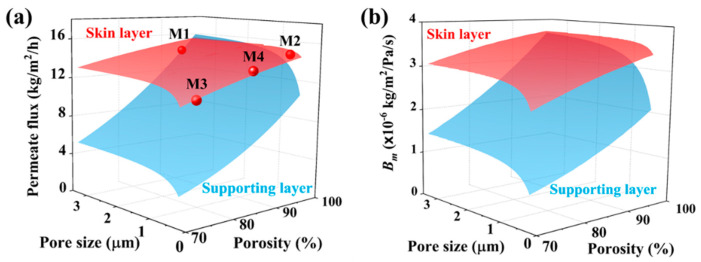
(**a**) Permeate flux and (**b**) *B_m_* for the membrane with the feed temperature at 70 °C. The porosity varies from 70% to 90%. The pore size varies from 0.2 μm to 3.3 μm. The skin layer parameters have an insignificant effect on the permeate flux. The transport behavior is governed by the supporting layer parameters.

As mentioned in Equation (6), the *B* value is determined by porosity, pore size, and thickness of each layer. Moreover, as mentioned in Equation (5), there is series connection of the mass transfer resistance between the skin layer and supporting layer. The influence of each layer on *B_m_* is not only determined by the relative change of *B*_1_ and *B*_2_ in the common range of pore size and porosity of each layer but also strongly influenced by the absolute value of *B*_1_ and *B*_2_. To find out the determining factor, *B_m_* based on the common ranges of porosity and pore size of each layer was estimated. To facilitate the analysis, the thicknesses of the skin layer and supporting layer are set as 7 μm and 50 μm for theoretical estimations, respectively. The module parameters and operation parameters of AGMD in this experiment are used in the estimations. The temperature of the feed and coolant are 70 °C and 20 °C, respectively. When estimating the influence of the skin layer parameters, the pore diameter of the supporting layer is 3.22 μm and the porosity is 93%. When estimating the influence of the supporting layer parameters, the skin layer parameters are set as the average values of M2, M3, and M4. The pore diameter is 0.4 μm, and the porosity is 85%. A commonly used stretched membrane and electrospinning membrane porosity range of 70%–95% and pore size range of 0.2–3.3 μm are adopted for estimations. The estimation process is based on our previous study [64]. The theoretical estimations are shown in Figure 10.

As shown in Figure 10a, the permeate flux varies from 11 kg/m^2^/h to15.7 kg/m^2^/h with the change of skin layer parameters, and the relative change is 42%. The permeate flux varies from 3 kg/m^2^/h to 16 kg/m^2^/h with the change of supporting layer parameters, and the relative change is 434%. Therefore, it can be considered that in the range of commonly used membrane parameters, the skin layer parameters have an insignificant effect on transport behavior. This is consistent with the experimental results as shown in Figure 8a: the MD with different membranes gives a similar permeate flux. This means that the skin layer only improves the LEP of the membrane (see Figure 7). As the main body of the membrane, the supporting layer not only improves the mechanical properties and thermal stability but also dominates the transport behavior. Modification and optimization of the internal structure of the supporting layer can realize the special application of MD and optimize the MD performance. The CFM provides a way for membrane with large pore size to be applied in MD without changing the MD performance.

The permeate flux can be characterized by *B_m_*. As shown in Figure 10b, *B_m_* varies from 2.44 × 10^−6^ kg/m^2^/Pa/s to 3.72 × 10^−6^ kg/m^2^/Pa/s with the change of skin layer parameters, and the relative change is 53%. *B_m_* varies from 0.78 × 10^−6^ kg/m^2^/Pa/s to 3.68 × 10^−6^ kg/m^2^/Pa/s with the change of supporting layer parameters, and the relative change is 373%. This indicates that the effects of skin layer parameters on *B_m_* can be ignored compared with the supporting layer parameters under the same conditions. With the change of skin layer parameters, the temperatures on both sides of the membrane are 69 ± 0.2 °C and 68 ± 0.1 °C, respectively, and the temperature difference across the membrane is 0.95 ± 0.03 °C (see SI Figure S4). With the change of the supporting layer parameters, the temperatures on both sides of the membrane are 69.3 ± 0.4 °C and 68.4 ± 0.5 °C, respectively, and the temperature difference across the membrane was 0.93 ± 0.16 °C. In AGMD, the skin layer and supporting layer parameters have an insignificant effect on the temperature distribution in the membrane. This is because the main thermal resistance in AGMD comes from the air gap.

Compared to the pore size, the porosity has a greater effect on *B_m_*. This is because *B_m_* increases squarely with the porosity according to Equation (6). The *B* value of Knudsen diffusion increases linearly with the pore size. Therefore, *B_m_* for M2 is the largest, and that for M3 is the smallest. This is consistent with the theoretical estimations. The relative change of *B_m_* is 13% with the pore size of the skin layer and 61% with that of the supporting layer under a constant porosity of 85%. The relative change of *B_m_* is 30% with the porosity of the skin layer and 165% with that of the supporting layer under a constant pore size of 0.4 μm.

As shown in Figure 10, the enhancement of permeate flux and *B_m_* decreases with the increasing pore size under a constant porosity. This is because the increase of *B* value of Knudsen diffusion decreases the influence of Knudsen diffusion according to Equation (6). The relative change of permeate flux is less than 15% when the pore size is larger than 1 μm. Increasing the pore size of the supporting layer can maximize the permeate flux and *B_m_*. When the pore size is larger than 15 μm, the transport mechanism in the supporting layer is molecular diffusion. Thus, the increase of pore size does not affect the permeate flux and *B_m_*. As shown in Figure 10a, when the pore size of the supporting layer is larger than 2.5 μm and the porosity is higher than 96%, the permeate flux of the CFM is greater than that of the M1 membrane. To achieve higher permeate flux, membranes with relatively higher porosity and larger pore size as the supporting layer is recommended based on our estimations.

## 4. Conclusions

The permeate flux and anti-wetting performance of MD can be improved simultaneously by the CFM. The CFMs can be fabricated by electrospinning a thin skin layer (7–9 μm) of PAN and PVP with various morphologies on a thick supporting layer (~50 μm) of PAN. The CFMs have good thermal stability, mechanical stability, and chemical stability. The skin layer increases the LEP of the CFM by 5 times. All the CFM gives a similar permeate flux. The skin layer and supporting layer parameters have an insignificant effect on the temperature distribution in the membrane. The permeate flux is mainly characterized by *B_m_*. The transport behavior is dominated by the supporting layer. Increasing the porosity can increase *B_m_* continuously and rapidly compared with pore size. Increasing the pore size to 1 μm can increase the penetrate flux rapidly. Continue to increase the pore size can reach the maximum permeate flux under a constant porosity. The optimized CFMs should have a supporting layer with high porosity and large pore size and a skin layer with high porosity and pore size less than 1 μm. This study provides a route of modifying many porous membranes for the application of MD.

## Figures and Tables

**Table 1:** Electrospinning parameters for polymer solutions and the resulting fiber diameter of membranes.

Materials	Flow Rate (mL/min)	Voltage (kV)	Rotating Speed (rpm)	Fiber Diameter (μm)
PAN (19 wt.%)	0.02	+16/−6	40	1.1 ± 0.1
PAN (11.5 wt.%)	0.02	+16/−6	40	0.2 ± 0.05
PAN/PVP (5.5 wt.%/6 wt.%)	0.02	+16/−6	40	0.25 ± 0.05
PAN (7 wt.%)	0.02	+12/−6	40	0.05 ± 0.02/0.6 ± 0.4 *

* The diameter of the fiber connecting the beads is 0.05 ± 0.02 μm. The bead diameter is 0.6 ± 0.4 μm.

**Table 2:** Parameters of the membranes.

Code of Sample Membranes	M1	M2	M3	M4
Supporting layer	PAN (19 wt.%)	PAN (19 wt.%)		
Skin layer		PAN (11.5 wt.%)	PAN/PVP (5.5 wt.%/6 wt.%)	PAN (7 wt.%)
*δ*_1_ (μm)	~7	~7	~9	~7
*δ_t_* (μm)	60 ± 2	64 ± 3	58 ± 5	62 ± 4
Contact angle (^o^)	151 ± 3	153 ± 3	155 ± 5	160 ± 3
LEP (kPa) (25 °C)	47	335	386	541
*ε_t_* (%)	93 ± 1	94 ± 1	90 ± 1	92 ± 1
*ε*_1_ (%)	~93	~97	~75	~86
*d*_1_ (μm)	3.22	0.49	0.42	0.27

See the nomenclature for abbreviations and codes.

## Supplementary Materials

The following are available online at https://www.mdpi.com/2077-0375/11/1/14/s1, Figure S1. FTIR spectra of untreated PAN powder, PVP powder, and skin layer of M3 after washing. Figure S2: (a) Morphology and (b) diameter distribution of the skin layer of M3 before washing. The average diameter is 0.19 ± 0.05 μm. Figure S3. Pressure drop of the membrane under the temperature in a range of 25 °C–80 °C. The flow rate is 0.3 mL/min. Figure S4. The temperature on both sides of the membrane (a) and temperature difference across the membrane (b) for the composite membrane with the feed temperature at 70 °C. *T_fm_* is the interface temperature between the feed and composite membrane. *T_ma_* is the interface temperature between the composite membrane and air gap.

## Nomenclature

*B*	mass transfer coefficient (kg/m^2^/s/Pa)
*C*	coefficients related to temperature in mass transfer diffusion (kg/m^2^/s/Pa)
*d*	pore diameter (μm)
*m*	mass (g)
*N*	permeate flux obtained from the test (kg/m^2^/h)
*P*	pressure (Pa)
*T*	temperature (°C)
Greek Letters	
*δ*	thickness (μm)
*ε*	porosity (%)
*ρ*	density (g/cm^3^)
Index	
*1*	skin layer
*2*	supporting layer
*a*	air
*c*	coolant
*f*	feed
*fm*	interface between the membrane and feed
*IPA*	isopropanol
*K*	Knudsen diffusion
*m*	membrane
*ma*	interface between the membrane and air gap
*M*	Molecular diffusion
*p*	polymer
*t*	total CFM
*wm*	membrane wetted with the isopropanol

## References

[1] Schofield R.-W., Fane A.G., Fell C.-J.-D. (1987). Heat and mass transfer in membrane distillation. J. Membr. Sci..

[2] Kimura S., Nakao S.-I., Shimatani S.-I. (1987). Transport phenomena in membrane distillation. J. Membr. Sci..

[3] Alkhudhiri A., Darwish N., Hilal N. (2012). Membrane distillation: A comprehensive review. Desalination.

[4] Drioli E., Ali A., Macedonio F. (2015). Membrane distillation: Recent developments and perspectives. Desalination.

[5] Lawson K.-W., Lloyd D.-R. (1997). Membrane distillation. J. Membr. Sci..

[6] Eykens L., De Sitter K., Dotremont C., Pinoy L., Van der Bruggen B. (2017). Membrane synthesis for membrane distillation: A review. Sep. Purif. Technol..

[7] Huang Y.-X., Wang Z., Jin J., Lin S. (2017). Novel Janus membrane for membrane distillation with simultaneous fouling and wetting resistance. Environ. Sci. Technol..

[8] Lalia B.-S., Guillen E., Arafat H.-A., Hashaikeh R. (2014). Nanocrystalline cellulose reinforced PVDF-HFP membranes for membrane distillation application. Desalination.

[9] Ali M.I., Summers E.-K., Arafat H.-A., Lienhard V.J.-H. (2012). Effects of membrane properties on water production cost in small scale membrane distillation systems. Desalination.

[10] Wang P., Chung T.-S. (2012). Design and fabrication of lotus-root-like multi-bore hollow fiber membrane for direct contact membrane distillation. J. Membr. Sci..

[11] Feng C., Khulbe K.-C., Matsuura T., Tabe S., Ismail A.-F. (2013). Preparation and characterization of electro-spun nanofiber membranes and their possible applications in water treatment. Sep. Purif. Technol..

[12] Khalifa A.-E. (2018). Performances of air gap and water gap MD desalination modules. Water Pract. Technol..

[13] He K., Hwang H.-J., Moon I.S. (2011). Air gap membrane distillation on the different types of membrane. Korean J. Chem. Eng..

[14] Tomaszewska M., Mientka A. (2009). Separation of HCl from HCl-H_2_SO_4_ solutions by membrane distillation. Desalination.

[15] Lalia B.-S., Guillen-Burrieza E., Arafat H.-A., Hashaikeh R. (2013). Fabrication and characterization of polyvinylidenefluoride-co-hexafluoropropylene (PVDF-HFP) electrospun membranes for direct contact membrane distillation. J. Membr. Sci..

[16] Jia W., Kharraz J.-A., Guo J., An A.-K. (2020). Superhydrophobic (polyvinylidene fluoride-co-hexafluoropropylene)/(polystyrene) composite membrane via a novel hybrid electrospin-electrospray process. J. Membr. Sci..

[17] Cai J., Liu X., Zhao Y., Guo F. (2018). Membrane desalination using surface fluorination treated electrospun polyacrylonitrile membranes with nonwoven structure and quasi-parallel fibrous structure. Desalination.

[18] Guo F., Servi A., Liu A., Gleason K.-K., Rutledge G.-C. (2015). Desalination by membrane distillation using electrospun polyamide fiber membranes with surface fluorination by chemical vapor deposition. ACS Appl. Mater. Interfaces.

[19] Adnan S., Hoang M., Wang H., Xie Z. (2012). Commercial PTFE membranes for membrane distillation application: Effect of microstructure and support material. Desalination.

[20] Khayet M., Wang R. (2018). Mixed matrix polytetrafluoroethylene/polysulfone electrospun nanofibrous membranes for water desalination by membrane distillation. ACS Appl. Mater. Interfaces.

[21] Xue J., Wu T., Dai Y., Xia Y. (2019). Electrospinning and electrospun nanofibers: Methods, materials, and applications. Chem. Rev..

[22] Fouladivanda M., Karimi-Sabet J., Abbasi F., Moosavian M.-A. (2021). Step-by-step improvement of mixed-matrix nanofiber membrane with functionalized graphene oxide for desalination via air-gap membrane distillation. Sep. Purif. Technol..

[23] Beauregard N., Al-Furaiji M., Dias G., Worthington M., Suresh A., Srivastava R., Burkey D.-D., McCutcheon J.-R. (2020). Enhancing iCVD modification of electrospun membranes for membrane distillation using a 3D printed Scaffold. Polymers.

[24] Tijing L.-D., Choi J.-S., Lee S., Kim S.-H., Shon H.-K. (2014). Recent progress of membrane distillation using electrospun nanofibrous membrane. J. Membr. Sci..

[25] Alkhudhiri A., Hilal N. (2017). Air gap membrane distillation: A detailed study of high saline solution. Desalination.

[26] Ahmed F.-E., Lalia B.-S., Hashaikeh R. (2015). A review on electrospinning for membrane fabrication: Challenges and applications. Desalination.

[27] Khayet M., García-Payo M.-C., García-Fernández L., Contreras-Martínez J. (2018). Dual-layered electrospun nanofibrous membranes for membrane distillation. Desalination.

[28] Attia H., Johnson D.-J., Wright C.-J., Hilal N. (2018). Comparison between dual-layer (superhydrophobic–hydrophobic) and single superhydrophobic layer electrospun membranes for heavy metal recovery by air-gap membrane distillation. Desalination.

[29] Tijing L.-D., Woo Y.-C., Johir M.-A.-H., Choi J.-S., Shon H.-K. (2014). A novel dual-layer bicomponent electrospun nanofibrous membrane for desalination by direct contact membrane distillation. Chem. Eng. J..

[30] Cheng D., Zhang J., Li N., Ng D., Gray S.-R., Xie Z. (2018). Antiwettability and performance stability of a composite hydrophobic/hydrophilic dual-layer membrane in wastewater treatment by membrane distillation. Ind. Eng. Chem. Res..

[31] Hou D., Wang Z., Wang K., Wang J., Lin S. (2018). Composite membrane with electrospun multiscale-textured surface for robust oil-fouling resistance in membrane distillation. J. Membr. Sci..

[32] Razmjou A., Arifin E., Dong G., Mansouri J., Chen V. (2012). Superhydrophobic modification of TiO_2_ nanocomposite PVDF membranes for applications in membrane distillation. J. Membr. Sci..

[33] Fan Y., Chen S., Zhao H., Liu Y. (2017). Distillation membrane constructed by TiO_2_ nanofiber followed by fluorination for excellent water desalination performance. Desalination.

[34] Li F., Huang J., Xia Q., Lou M., Yang B., Tian Q., Liu Y. (2018). Direct contact membrane distillation for the treatment of industrial dyeing wastewater and characteristic pollutants. Sep. Purif. Technol..

[35] Liao Y., Loh C.-H., Wang R., Fane A.-G. (2014). Electrospun superhydrophobic membranes with unique structures for membrane distillation. ACS Appl. Mater. Interfaces.

[36] Zhu Z., Liu Z., Zhong L., Song C., Shi W., Cui F., Wang W. (2018). Breathable and asymmetrically superwettable Janus membrane with robust oil-fouling resistance for durable membrane distillation. J. Membr. Sci..

[37] Humoud M.-S., Roy S., Mitra S. (2020). Enhanced performance of carbon nanotube immobilized membrane for the treatment of high salinity produced water via direct contact membrane distillation. Membranes.

[38] Gethard K., Sae-Khow O., Mitra S. (2011). Water desalination using carbon-nanotube-enhanced membrane distillation. ACS Appl. Mater. Interfaces.

[39] Tan Y.-Z., Ang E.-H., Chew J.-W. (2019). Metallic spacers to enhance membrane distillation. J. Membr. Sci..

[40] Liao Y., Wang R., Fane A.-G. (2013). Engineering superhydrophobic surface on poly(vinylidene fluoride) nanofiber membranes for direct contact membrane distillation. J. Membr. Sci..

[41] An X., Xu G., Xie B., Hu Y. (2019). Structural tailoring of hierarchical fibrous composite membranes to balance mass transfer and heat transfer for state-of-the-art desalination performance in membrane distillation. J. Mater. Chem. A.

[42] Essalhi M., Khayet M. (2012). Surface segregation of fluorinated modifying macromolecule for hydrophobic/hydrophilic membrane preparation and application in air gap and direct contact membrane distillation. J. Membr. Sci..

[43] Bonyadi S., Chung T.-S. (2007). Flux enhancement in membrane distillation by fabrication of dual layer hydrophilic-hydrophobic hollow fiber membranes. J. Membr. Sci..

[44] Woo Y.-C., Tijing L.-D., Park M.-J., Yao M., Choi J.-S., Lee S., Kim S.-H., An K.-J., Shon H.-K. (2017). Electrospun dual-layer nonwoven membrane for desalination by air gap membrane distillation. Desalination.

[45] Chew N.-G.-P., Zhang Y., Goh K., Ho J.-S., Xu R., Wang R. (2019). Hierarchically structured Janus membrane surfaces for enhanced membrane distillation performance. ACS Appl. Mater. Interfaces.

[46] Shaulsky E., Nejati S., Boo C., Perreault F., Osuji C.-O., Elimelech M. (2017). Post-fabrication modification of electrospun nanofiber mats with polymer coating for membrane distillation applications. J. Membr. Sci..

[47] Cai J., Guo F. (2017). Study of mass transfer coefficient in membrane desalination. Desalination.

[48] Young T. (1805). An essay on the cohesion of fluids. Philos. Trans. R. Soc. Lond..

[49] Cong S., Guo F. (2019). Janus nanofibrous membranes for desalination by air gap membrane distillation. ACS Appl. Polym. Mater..

[50] Darcy H. (1856). Les Fontaines Publiques de la Ville de Dijon: Exposition et Application.

[51] Bindels M., Brand N., Nelemans B. (2018). Modeling of semibatch air gap membrane distillation. Desalination.

[52] Cong S., Liu X., Guo F. (2019). Membrane distillation using surface modified multi-layer porous ceramics. Int. J. Heat Mass Transf..

[53] Knudsen M. (1909). Die Gesetze der molekularströmung und der inneren reibungsströmung der gase durch röhren. Ann. Phys..

[54] Fick A. (1855). Ueber Diffusion. Ann. Phys..

[55] Homaeigohar S., Koll J., Lilleodden E.-T., Elbahri M. (2012). The solvent induced interfiber adhesion and its influence on the mechanical and filtration properties of polyethersulfone electrospun nanofibrous microfiltration membranes. Sep. Purif. Technol..

[56] Soliman S., Sant S., Nichol J.-W., Khabiry M., Traversa E., Khademhosseini A. (2011). Controlling the porosity of fibrous scaffolds by modulating the fiber diameter and packing density. J. Biomed. Mater. Res. Part A.

[57] Cassie A.-B.-D., Baxter S. (1944). Wettability of porous surfaces. Trans. Faraday Soc..

[58] Park I.-J., Lee S.-B., Choi C.-K., Kim K.-J. (1996). Surface properties and structure of poly(perfluoroalkylethyl methacrylate). J. Colloid Interface Sci..

[59] Ma M., Mao Y., Gupta M., Gleason K.-K., Rutledge G.-C. (2005). Superhydrophobic fabrics produced by electrospinning and chemical vapor deposition. Macromolecules.

[60] Li K., Hou D., Fu C., Wang K., Wang J. (2019). Fabrication of PVDF nanofibrous hydrophobic composite membranes reinforced with fabric substrates via electrospinning for membrane distillation desalination. J. Environ. Sci..

[61] Essalhi M., Khayet M. (2014). Self-sustained webs of polyvinylidene fluoride electrospun nano-fibers: Effects of polymer concentration and desalination by direct contact membrane distillation. J. Membr. Sci..

[62] Bagherzadeh R., Najar S.-S., Latifi M., Tehran M.-A., Kong L. (2013). A theoretical analysis and prediction of pore size and pore size distribution in electrospun multilayer nanofibrous materials. J. Biomed. Mater. Res. Part A.

[63] Hottel H.-C., Noble J.-J., Sarofim A.-F., Silcox G.-D., Wankat P.-C., Knaebel K.-S. (2008). Perry’s Chemical Engineers’ Handbook: Heat and Mass Transfer.

[64] Cai J., Yin H., Guo F. (2020). Transport analysis of material gap membrane distillation desalination processes. Desalination.

