# Supplementary material for: Transport Analysis of Anti-Wetting Composite Fibrous Membranes for Membrane Distillation
Source: Membranes (Basel). 2020 Dec 24;11(1):14. doi: 10.3390/membranes11010014 (PMC7823856; doi:10.3390/membranes11010014)
Supplement: Supplementary file 1 [file membranes-11-00014-s001.pdf]

membranes

MDPI

Supplementary Materials: Transport Analysis of Anti-Wetting Composite Fibrous Membranes for Membrane Distillation

Jingcheng Cai, Zeman Liu, Fei Guo *

School of Energy and Power Engineering, Key Laboratory of Ocean Energy Utilization and Energy Conservation of Ministry of Education, Dalian University of Technology, No.2 Linggong Road, Dalian 116024, China

* Correspondence: feiguo@dlut.edu.cn

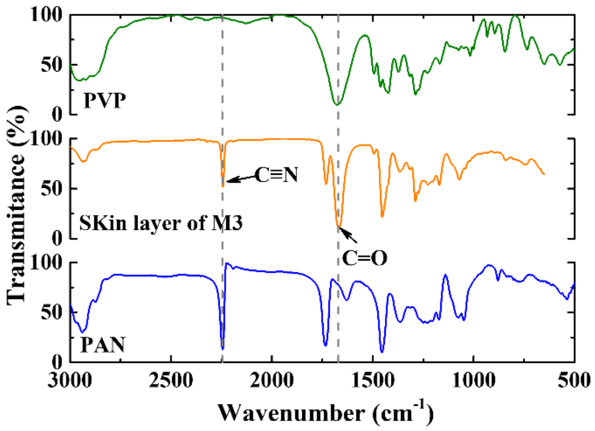

Figure S1. FTIR spectra of untreated PAN powder, PVP powder, and skin layer of M3 after washing.

Flourier-transform infrared spectroscopy (FTIR) analysis was further carried out. The peak at 2241 cm^{-1} could be ascribed to the bending vibration of C≡N, while that at 1667 cm^{-1} could be assigned to the stretching vibration of C=O. The surface layer of M3 contains the functional group of PAN (C≡N) and the functional group of PVP (C=O). Thus, PVP is adsorbed on the fiber surface.

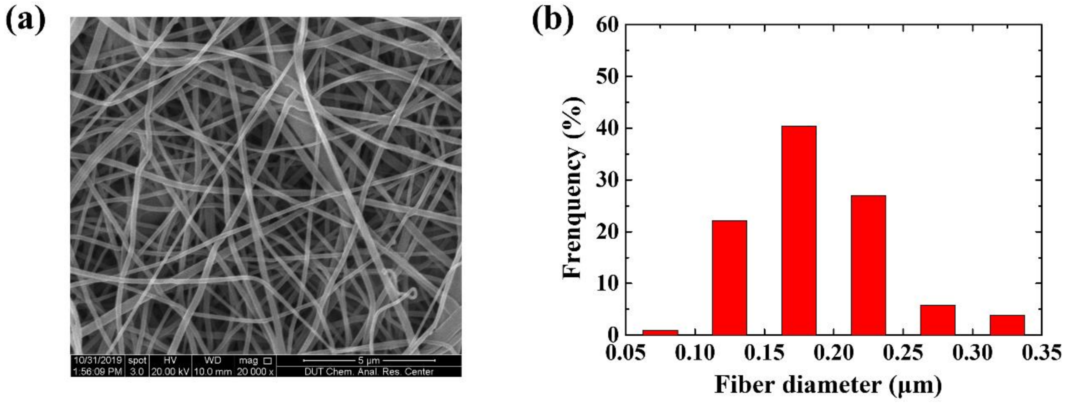

Figure S2. (**a**) Morphology and (**b**) diameter distribution of the skin layer of M3 before washing. The average diameter is 0.19 ± 0.05 μm.

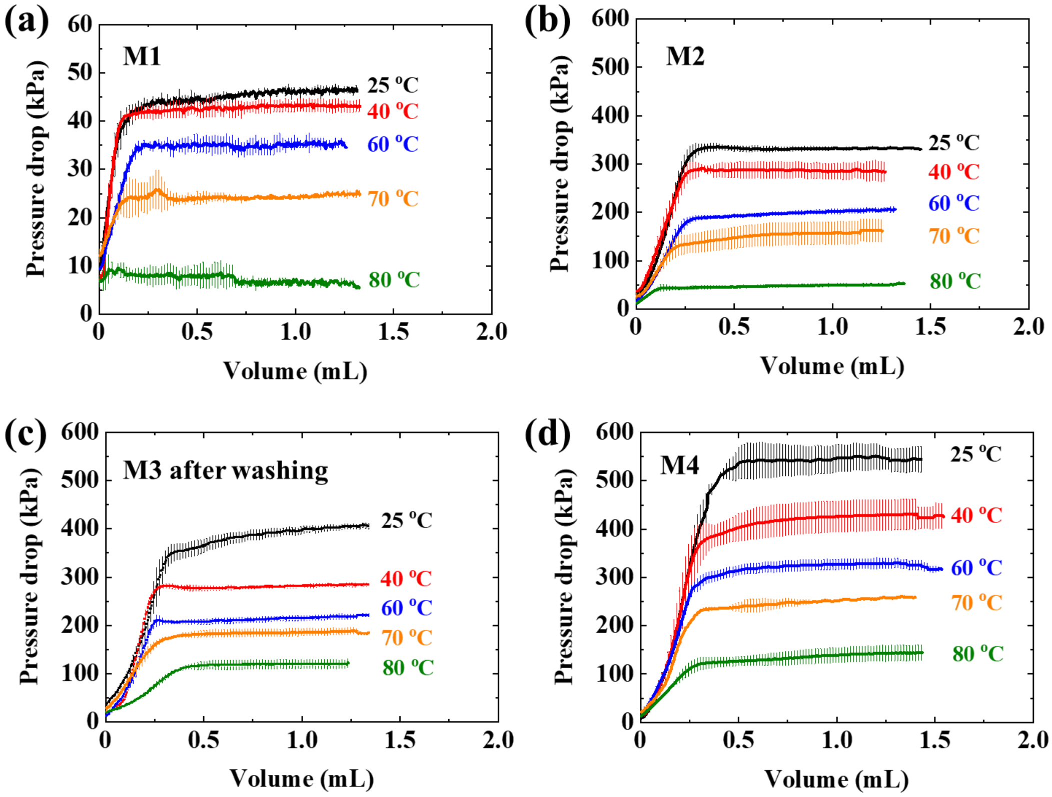

Figure S3. Dynamic recording of the pressure drop of the membranes in this work. (**a**) M1, (**b**) M2, (**c**) M3 after washing, (**d**) M4. The test water flow rate was maintained at 0.3 mL/min. The temperature of the test water flow was tuned from 25 to 80 °C by a heating wire wounded on the test syringe.

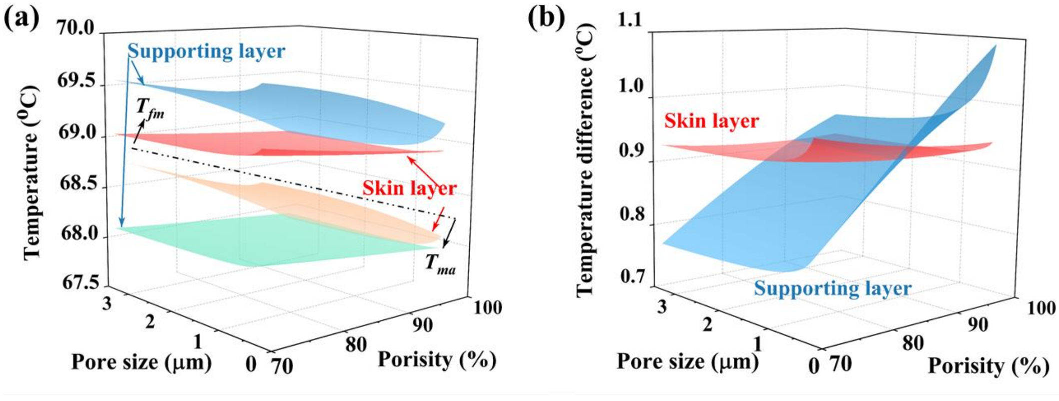

Figure S4. The temperature on both sides of the membrane (**a**) and temperature difference across the membrane (**b**) for the composite membrane with the feed temperature at 70 °C. T_{fm} is the interface temperature between the feed and composite membrane. T_{ma} is the interface temperature between the composite membrane and air gap.
